# Development of an embedded multimodality imaging platform for onco-pharmacology using a smart anticancer prodrug as an example

**DOI:** 10.1038/s41598-020-59561-8

**Published:** 2020-02-14

**Authors:** Florian Raes, Serigne Moussa Badiane, Brigitte Renoux, Sébastien Papot, Stéphanie Lerondel, Alain Le Pape

**Affiliations:** 1 Institute for Cancer Research, ICR, Sutton, London, United Kingdom; 2 Centre for Small Animal Imaging, CIPA, Phenomin-TAAM CNRS UPS44, Orléans, France; 3 UFR des Sciences de la Santé, Université Gaston Berger, Saint-Louis, Senegal; 4 IC2MP, Chemistry Institute of Poitiers: Materials and Natural Ressources; Programmed Molecular Systems Team, UMR-CNRS 7285, Poitiers, France

**Keywords:** Cancer imaging, Cancer models, Cancer therapy

## Abstract

Increasingly, *in vivo* imaging holds a strategic position in bio-pharmaceutical innovation. We will present the implementation of an integrated multimodal imaging setup enabling the assessment of multiple, complementary parameters. The system allows the fusion of information provided by: Near infrared fluorescent biomarkers, bioluminescence (for tumor proliferation status), Photoacoustic and Ultrasound imaging. We will study representative applications to the development of a smart prodrug, delivering a highly cytotoxic chemotherapeutic agent to cancer tumors. The results realized the ability of this embedded, multimodality imaging platform to firstly detect bioluminescent and fluorescent signals, and secondly, record ultrasound and photoacoustic data from the same animal. This study demonstrated that the prodrug was effective in three different models of hypoxia in human cancers compared to the parental cytotoxic agent and the vehicle groups. Monitoring by photoacoustic imaging during the treatments revealed that the prodrug exhibits an intrinsic capability to prevent the progression of tumor hypoxia. It is essential for onco-pharmacology studies to precisely document the hypoxic status of tumors both before and during the time course of treatments. This approach opens new perspectives for exploitation of preclinical mouse models of cancer, especially when considering associations between hypoxia, neoangiogenesis and antitumor activity.

## Introduction

Preclinical imaging plays a key role in translational research, particularly for early cancer detection and the assessment of therapeutic agents and their efficacy^1,2^. The first generation of preclinical imaging strategies dedicated to assessments in murine cancer models derived from medical imaging modalities. These include: Computed Tomography (CT), Positron Emission Tomography (PET), Single Photon Emission Computed Tomography (SPECT) and Magnetic Resonance Imaging (MRI). However, regarding the use of both CT and radioactive techniques, the dosimetry resulting from repeated irradiations received by animals (including tumors) have to be considered when imaging sessions are performed longitudinally.

Molecular imaging modalities such as bioluminescence (BLI), near infrared fluorescence (NIRF), ultrasound imaging (US) and recently photoacoustic imaging (PA) are becoming increasingly useful imaging modalities for preclinical research. Their use provides relevant information regarding tumor kinetics and require no particular radioprotection environment compared to nuclear techniques. Further, unlike CT imaging, these modalities are inert when regarding tumor growth. Implementing US, PA, BLI and NIRF conducted to reconsider the translational research overcoming the limitations resulting in possible irrelevant data.

**Figure 1:**
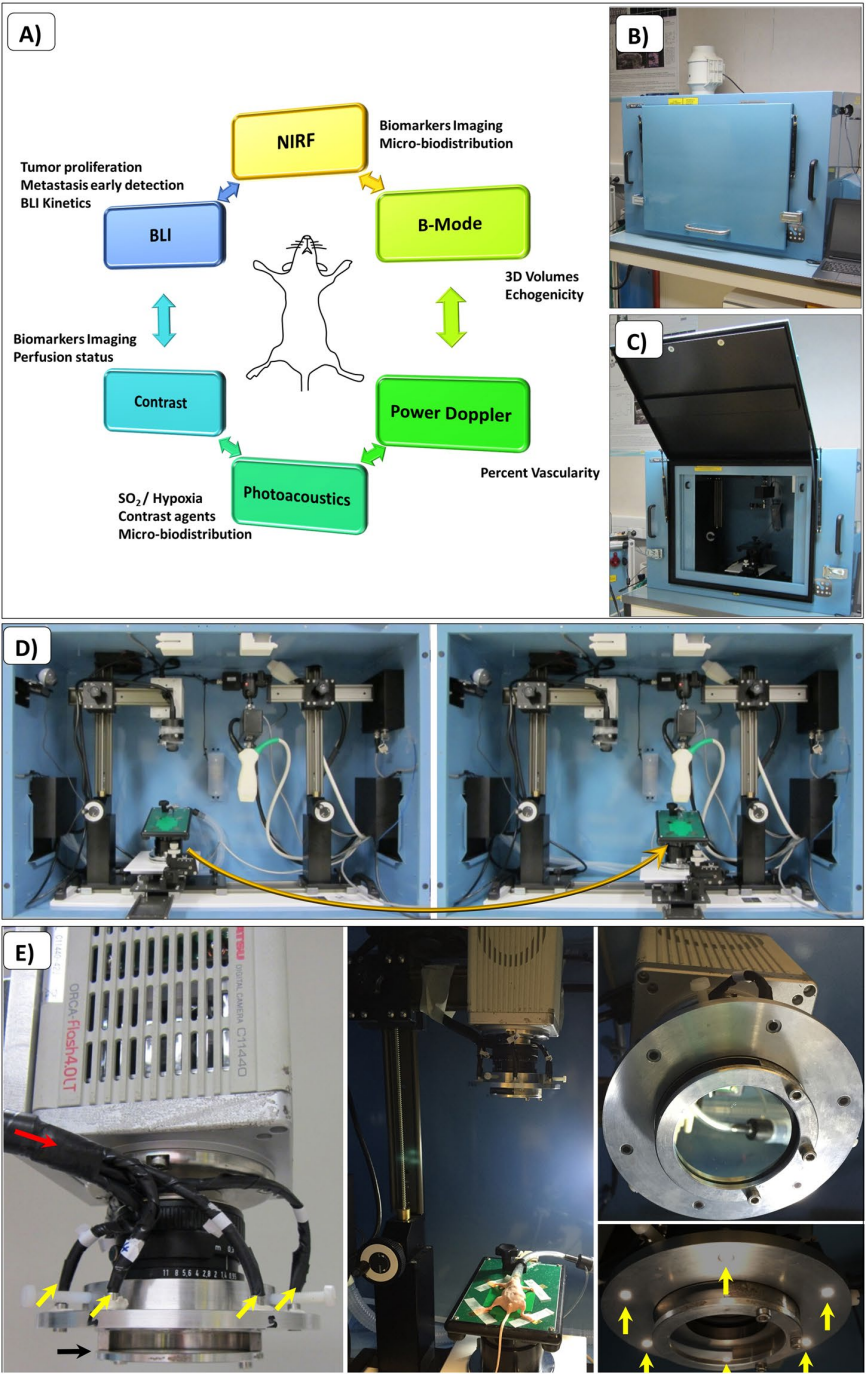
Set up of the embedded multimodality imaging plateform. (**A**) Rationale for the selection of imaging modalities for each dedicated protocol. (**B**) Modified light enclosure dark box, door closed. (**C**) Door opened. (**D**) The first step is the BLI and NIRF configuration, with the camera, then the second step is US and PA configuration, with the ultrasound/photoacoustic transducer. (**E**) NIRF illumination device. The camera owns a lens with a filter holder in order to change emission filters allowing the assessment of fluorochromes with different emission spectra. The filtered fluorescence sources of light are positioned in six points around the lens (yellow arrows).

In this work, we propose a quadri-modal imaging strategy, bringing together biophotonics (e.g. BLI and NIRF), US and PA in an embedded platform (Fig. 1A–D). Based on recent literature, this combination has never been described before. This prototype platform consists of a new-generation, low-cost CMOS camera (Fig. 1E) for BLI and NIRF, inserted inside the commercial enclosure of the VEVO LAZR system (US and PA acquisitions). Data obtained using this platform provide complementary information from each modality in a two-step procedure within only one anesthesia. Unlike current laboratory setups, multiple imaging parameters can be measured without the need to transfer animals between different preclinical imaging systems.

Unlike clinical imaging modalities, optical imaging in small animals allows a large variety of non-invasive *in vivo* investigations such as the receptor expression status, evaluation of apoptosis or necrosis, early cell localization, real-time signaling pathway activity, angiogenesis, tumor cell tracking, drug response, bone remodelling, invasion or proteolysis assessments. It is applicable to any tumor site, from the primary tumor site to the detection of metastasis with a longitudinal monitoring. Furthermore, biophotonics techniques are cheap, with affordable consumables (e.g. targeted probes, luciferin, antibodies, as compared to isotopes) and a very low maintainance cost.

Multimodal imaging is advantageous in preclinical research as it can allow for the identification, development and improvement of drug candidates thanks to the accurate detection of compounds efficacy in animals with both high sensitivity and resolution. Moreover, multimodal imaging accelerates the development of new imaging markers and improves our understanding of cancer’s pathological processes thanks to the complementarity of anatomical and molecular data^3,4^. BLI has the advantage of being highly sensitive with low background noise disturbances and NIRF imaging presents the advantage to be highly specific when implementing targeted fluorophores^5^. The panel of contrast agents and targeted probes available in preclinical research is impressive as compared to clinical research, making preclinical investigations of great importance in identifying the pathognomonic changes of the cancer disease.

Owing to the use of orthotopic models of human cancer for drug development, and therefore deep tumor location in such cancer models, the embedded platform requires an associated anatomical imaging modality for the assessment of the antitumor activity^6^. US is a high throughput imaging modality, adaptable and very useful for anatomical screening of organ disease and pathology^7,8^. Contrast enhanced ultrasound imaging (CEUS) is gaining popularity in both the preclinical and clinical fields. The use of CEUS dedicated contrast agents (biocompatible microbubbles (MBs) consisting of a gas core surrounded by a lipid shell) can detect very slow blood flows with a high sensitivity. Targeted MBs through vascular biomarkers can enable quantification of specific parameters including some biomarkers’ expression through measuring differential Targeted Enhancement (dTE). Ultrasound Molecular Imaging (USMI) utilizing molecularly targeted MBs enables the characterization of patho-physiological processes at a molecular level^2,9^, such as a work from *Cai et al*. investigating VEGFR2 targeted MBs using these techniques^10^. MBs can also be targeted through different vascular biomarkers such as αVβ3 integrins, VEGF, VCAM, MadCAM, glycoprotein IIb/IIIa or endoglin^11,12^. Multimodal contrast agents implementing the use of microbubbles are currently being developed, including the development of MBs filled with fluorescent dyes that can not only be used as contrast agents for US, but also as PA or NIRF^13^.

US and PA utilize complementary and synergistic instrumentation (i.e. ultrasound transducer) providing a real-time combination of anatomical, functional and molecular data in a single image acquisition^14^. Because of the existing differences in optical absorption between oxy-haemoglobin and deoxy-haemoglobin, PA of haemoglobin enables both functional and anatomical imaging of vascular structures and provides the percentage of haemoglobin saturation with oxygen (SO_2_)^15^. PA imaging can accurately map areas of hypoxia within tumors so that hypoxic volumes can be documented^16,17^. Latest USMI and PA approaches are mostly available in small animals as their use require either targeted agents and contrast agents that are not yet approved in clinical research, or dedicated transducers that are currently under development. Therefore, the main advantage of small animal USMI and PA in this platform is the access to a wide range of molecular targets, functional analysis as well as basic measurements (e.g. volume, function, anatomy, morphology, characterize tissues, receptor expression …).

Hypoxia is an important parameter that has to be monitored as it is a key determinant of outcome for human cancers^18^. Furthermore, hypoxia is of major concern for the development and validation of both hypoxia-targeted and hypoxia-modification therapies^19^. Because hypoxia is associated with increased tumor aggressiveness, understanding the type of hypoxia (acute hypoxia due to transient hypo-perfusion versus chronic hypoxia due to a chaotic tumor vasculature) could enable clinicians in making decisions in the management of therapies^20^.

The experimental design for this work was to implement a multimodal imaging approach for applications to onco-pharmacology. As an example, a novel prodrug (a β-Glucuronidase-Responsive Albumin-Binding Prodrug of MMAE) was studied in several experimental cancer models^21,22^. The embedded multimodal imaging platform was used to investigate therapeutic effects on both superficial and orthotopic tumor models, in order to provide essential information on a wide range of specific tumor growth parameters. This approach allows demonstrating the outstanding efficacy of this prodrug while showing intrinsic capability to prevent the progression of tumor hypoxia during tumor growth.

## Materials and Methods

### Ethics statement

All animals procedures were performed in accordance with European ethical guidelines (European directives 2010/63/EU) and were approved by the Regional Committee for Animal Care and Ethics in Animal Experiments (C2EA-03 Comité d’éthique en expérimentation animale Campus CNRS d’Orléans).

### Cell culture

MDA-MB-231-luc2 human triple-negative breast adenocarcinoma cell line was obtained from Perkin Elmer (France). KB human epithelial cancer cell line and Mia-PaCa2 human ductal pancreatic adenocarcinoma cell line were obtained from ATCC and transfected to express the luciferase (performed by V. Trichet for Mia-PaCa-2 cells, INSERM U957 EA3822 Nantes; performed by J. Clarhaut for KB cells, IC2MP UMR7285 Poitiers). Cancer cell lines were maintained according to the supplier’s instructions.

### Animals

Pathogen-free 6 to 8 weeks-old female nude Balb/c and Swiss mice were purchased from Charles River Laboratories (France). Mice were acclimatized for 7 days in the laboratory before experimentation and were maintained in sterilized filter-stopped cages inside a controlled ventilated rack with access to food and water *ad libitum*. They were examined daily for clinical signs, distress, decreased physical activity and weighed 3 times a week. Each animal serves as its own control, thereby increasing the accuracy of experiments.

### Cells xenografts

#### Orthotopic pancreatic Mia-PaCa2 xenografts

Xenografts from the pancreatic cancer cell line Mia-PaCa2 were established in Swiss nude mice by orthotopic implantation. Mice were anesthetized by inhalation of 1.5% isoflurane with air (Isoflo®, AXIENCE S.A.S, Pantin, France). Abdomens of mice were prepared with a solution of povidone iodine (Betadine®, ASTA Medica, Belgium). A small transverse incision was made in the left lateral flank through the skin and peritoneum. The tip of pancreatic tail was gently grasped and pancreas/spleen were externalized in a lateral direction to be fully exposed. The inoculum (2 × 10^6^ Mia-PaCa2 cells in 10 μL of PBS) was slowly injected using a 27-gauge needle of a Hamilton syringe (Hamilton, Bonaduz, Switzerland). The spleen was then repositioned in the abdominal cavity, and 7-0 and 5-0 sutures used to close the peritoneum and skin, respectively.

#### Orthotopic breast MDA-MB-231 xenografts

Xenografts from the breast cancer cell line MDA-MB-231-luc2 were established in Balb/c nude mice by orthotopic implantation. Mice were anaesthetized by inhalation of 1.5% isoflurane with air (Isoflo®, AXIENCE S.A.S, Pantin, France). The inoculum (2 × 10^6^ tumor cells in 100 μL PBS) was injected in the mammary fat pads of animals.

#### Subcutaneous KB xenografts

Xenografts from the KB human oral epithelial cancer cell line were established in BALB/c Nude mice by subcutaneous implantation. Mice were anesthetized by inhalation of 1.5% isoflurane with air. The inoculum (1 × 10^6^ tumor cells in 150 μL of a 50:50 Matrigel/PBS mix) was injected in the dorsal flank of animals.

### Bioluminescence and near infrared fluorescence imaging

A miniaturized ORCA Flash 4.0 LT camera (Hamamatsu photonics) was implemented for BLI and NIRF acquisitions in the embedded platform. The field of view is large enough to allow a full body imaging of mice. The maximum resolution is 2048 × 2048 pixels and the size of the CMOS sensor is 13.3 × 13.3 mm with 6.5 × 6.5 µm cells. Grayscale reference images were obtained with a white light illumination. The readout speed of the camera is 30 frames/s at the full resolution. Technical specifications are given in Table 1. The new generation CMOS device allowed for BLI recordings within 10 seconds/frames allowing kinetic studies in animal models with metastases. NIRF acquisitions were performed using two seconds/frames allowing for well delineated foci for superficial tumors, with a sensitivity up to 800 nm (Quantum efficiency of the camera = 40). For NIRF imaging, we used dedicated interchangeable filters for the emission, allowing imaging of fluorophores absorbing light at 680 nm (Excitation: 640 ± 50 nm; Emission: 740 ± 80 nm) or 750 nm (Excitation: 748 ± 37 nm; Emission: 843 ± 33 nm). The Solos Endoscopy GS9250 ELS-2 Xenon Arc Automatic Light Source (Solos Endoscopy Inc., Boston, USA) excitation source of light was used for both basic reference grayscale images as well as filtered excitation illumination of animals. In order to simultaneously monitor the expression of two parameters with specific probes, we applied a multi-wavelength strategy achieving the use of commutable filters for both emission and excitation. We further implemented a more convenient procedure directly based on the use of the mono-wavelength from the PA laser with advantage of a better discrimination between excitation and emission spectra.

**Table 1:** Technical specifications of the different modalities implemented in the platform.

		Type of modality	Spatial Resolution			Depth	Field of view	Temporal resolution/Throughput	kinetics
			axial	lateral	transverse				
Optical	BLI	2D	70 µm	70 µm	N.A.	~2 cm	14 cm × 14 cm	seconds (generally 10 seconds for a basic acquisition)	☑
	NIRF	2D	70 µm	70 µm	N.A.	~1 cm	14 cm × 14 cm	seconds (generally 2 seconds for a basic acquisition)	☑
US/PA	US	3D	75 µm*	165 µm*	from 30 µm using the stepper motor*	~3 cm	23,1 mm × 36 mm	minutes (from 1 min to 25 min depending on the type of acquisition)	☑
	PA	3D	75 µm*	165 µm*	from 30 µm using the stepper motor*	~2 cm	23,1 mm × 36 mm	minutes (from 5 min to 25 min depending on the type of acquisition)	☑

*Spatial resolutions given using the bi-modal transducer owning a 21 MHz central frequency.

In order to validate the results obtained with both BLI and NIRF, we used an IVIS-Lumina II (Perkin Elmer, France) generating a pseudo-colored image representing light intensity superimposed over a greyscale reference image. Acquisition binning and duration were set depending on tumor activity. Signal intensity was quantified as the total flux (photons/seconds) within ROIs drawn manually using Living Image 4.4 software (Perkin Elmer, France). When two different fluorescent probes were used in a same animal, an automated NIRF spectral unmixing procedure enabled the differentiation of signal from each probe. The automated spectral unmixing method used by the IVIS software is based on a Multivariate Curve Resolution Alternating Least-Square Scheme.

Anesthetized mice (by inhalation of 1.5% isoflurane with air (Isoflo®, AXIENCE S.A.S, Pantin, France) were placed on the thermostatically controlled heating pad from the ultrasound or from the Perkin Elmer system, in a supine position (for breast and pancreatic cancer models) or in prone position (for the Head and Neck cancer model). Paws of animals were secured to the Vevo pad through four electrocardiography leads. Each mouse was IP injected with 100 mg/kg luciferin potassium salt (Promega, France). Regarding the NIRF probes, 3 different dyes have been used, the ICG, the AF750 and the IR-Dye800. For NIRF image acquisitions, animals received an IV injection of a monoclonal antibody labelled either with ICG-NHS (Intrace Medical), AF750-NHS (SAIVI kit, Invitrogen) or IR-Dye800-NHS (LI-COR) 24 H before acquisitions protocols. For the labelling process, a 5 mg/mL cetuximab solution (Roche Laboratories) was used, with a post labelling concentration adjusted to 1 mg/mL. Dyes were conjugated using the reactivity of their NHS-ester groups and amine-reactive groups from the antibodies. Animals received 50 µL of the solution. These 3 fluorochromes own different fluorescent properties (ICG λ_ex_: 780 nm; λ_em_: 812 nm//AF750 λ_ex_: 749 nm; λ_em_: 755 nm//IR-Dye800 λ_ex_: 767 nm; λ_em_: 791 nm). These different fluorochromes were implemented here in order to evaluate their detectability by NIRF and their potential as PA contrast agents.

### Ultrasound imaging

Anesthetized mice were placed on the VEVO pad. Respiratory gating allows avoiding artefacts due to respiratory movements of the animal. Temperature of the animals was recorded with an internal temperature probe. An aqueous warmed ultrasonic gel (Supragel®, LCH, France) was applied on the skin to optimize the visualization of internal organs. Tumors were imaged with the VisualSonics Vevo™ LAZR (Fujifilm VisualSonics Inc, Toronto, Canada). A transducer with central frequency at 40 MHz, providing axial resolution of 40 μm with a 14.1 mm × 15 mm field of view, was used for imaging of smaller tumors. A transducer with central frequency at 21 MHz transducer, providing axial resolution of 75 μm with a 23.1 mm × 36 mm field of view, was used for larger tumor imaging. 3D scans of ultrasound image were recorded digitally and reviewed. Tumor areas in a coronal plane was measured by manually tracing the margin of tumors using VEVO LAB software. The software then calculated volumes enclosed within the delineated region on each coronal plane slice acquired. Tumor perfusion status and VEGFR2 expression were assessed by contrast enhanced ultrasound (CEUS) imaging following IV injection (tail vein) of Vevo MicroMarker™ and Target-Ready MicroMarker™ coupled with either anti-VEGFR2 or isotype control antibodies (eBioscience, USA). Imaging protocols were performed with the destruction-replenishment sequences. Data were processed with the VevoCQ™ software, and parameters such as Peak Enhancement (PE), relative Blood Volume (rBV), Wash-in Rate (WiR), and Wash-in Area Under the Curve (WiAUC) were obtained. The instrument is calibrated to allow measurements to be determined accurately. Technical specifications are given in Table 1.

### Photoacoustic imaging

During PA acquisitions, gain, power of the laser, depth of imaging, frame averaging and frame rate were kept constant, so that every data sets were acquired homogeneously. With the aim to differentiate the signal from injected probes (ICG, IR-Dye800 and AF750) compared to endogenous signal, a multi-spectral analysis and correction was performed. A pixel to pixel analysis was achieved with a comparison to an already recorded spectra library (Fig. S1). Average SO_2_ and 3D SO_2_ were monitored, and hypoxic areas/volumes documented, in 2D and 3D using the dedicated Oxy-Hemo mode. The total content of haemoglobin (HbT) could also be extracted from these data. In order to delineate hypoxic regions within tumors, we set a display threshold on the PA color scale to determine the hypoxic volumes in a reproducible manner (black to blue colors were set as <14% of SO_2_ and white to red colors were set as >14% of SO_2_ so that hypoxic ROIs could be easily delineated). This specific threshold has been used to delineate the hypoxic areas on each slice of the 3D and 2D acquisitions. Technical specifications are given in Table 1.

### Co-registration of data obtained from both camera and transducer

The co-registration of acquisitions from both biophotonics and ultrasounds was performed using advanced imaging analysis software (ImageJ, v1.50b). Data obtained were digitally fused in order to match the size of each imaging modality. Coronal plane’s 3D render from 3D US and PA acquisitions were merged on 2D acquisition, so that it was possible to compare the data. External landmarks (the shape of each animal’s body) were used to merge images from the US/PA onto images from biophotonics. Images were resized using a defined factor. 3D renders were then perfectly merged onto 2D acquisitions from BLI and NIRF.

### Hypoxia immunostaining

Immunostaining was performed with the Hypoxyprobe kit (Hypoxyprobe, Burlington, USA), following the supplier’s instructions. Mice received IV injection of 120 mg/kg of the pimonidazole solution and sacrificed 60 minutes following injection. Tumors were then resected and fixed for 24 H in forlmaldehyde (10%). Tumors embedded in paraffin were sectioned (7 µm) for alternate antipimonidazole and hematoxylin/eosin staining. A mouse anti-pimonidazole antibody (Abcam, Cambridge, USA) was used as a primary antibody in order to label pimonidazole adducts. A goat FITC-labelled anti-mouse antibody (Abcam, Cambridge, USA) was then used as a secondary antibody. The hematoxylin/eosin staining was performed (by Novaxia, Saint-Laurent-Nouan, France) to investigate areas of necrosis as compared to hypoxic regions.

### Therapeutic scheme

#### Breast tumor model

14 days after cells injection mice received either Prodrug 1 (4 mg/kg), MMAE (positive control; 0.5 mg/kg; MTD in BALB/C mice) or vehicle (DMSO:PBS 5%:95%), intravenously, once a week for five weeks (tumors size measured by 3D US on the day the treatments started: Vehicle 7.62 ± 1.16 mm^3^, MMAE 8.6 ± 1.17 mm^3^, BR-Alb 8.69 ± 0.86 mm^3^ (n = 6)).

#### Pancreatic tumor model

7 days after cells injections mice received either Prodrug 1 (4 mg/kg), MMAE (positive control; 0.3 mg/kg; MTD in Swiss mice) or vehicle (DMSO:PBS 5%:95%), intravenously, once a week for three weeks (tumors size measured by 3D US on the day the treatments started: Vehicle 55.52 ± 2.5 mm^3^, MMAE 72.28 ± 4.7 mm^3^, BR-Alb 76.42 ± 6.8 mm^3^ (n = 6)).

#### Head and neck tumor model

7 days after cells injection mice received either Prodrug 1 (2 mg/kg), MMAE (positive control; 0.5 mg/kg; MTD in BALB/C mice) or vehicle (DMSO:PBS 5%:95%), intravenously, once a week for four weeks. A lower dose of Prodrug 1 was chosen to determine development of hypoxic regions in this model (tumors size measured by 3D US on the day the treatments started: Vehicle 50.29 ± 9.4 mm^3^, MMAE 51.70 ± 9.75 mm^3^, BR-Alb 49.27 ± 9.77 mm^3^ (n = 8)) (prodrug 1 effects were already published, *Renoux et al*. 2017).

### Sacrifice

Mice under anesthesia were sacrificed by cervical dislocation (as recommended by ethics procedures) and tumors were collected from each animal for further *ex vivo* assessments.

### Statistical analysis

Statistical analysis was performed using GraphPad Prism software 5.0 (GraphPad, San Diego, USA). A two-way repeated-measure analysis of variance followed by Bonferroni post-tests was used for all the data of over time course. Differences were considered significant at p < 0.05. An unpaired student t test was performed for the comparisons between punctual data points. Differences were considered significant at p < 0.05.

## Results

### Utilization of the embedded multimodal imaging platform

#### Functional characterization of a tumor

First of all, we validated our targeted fluorescent probes. We assessed NIRF signals from cetuximab (antibody targeting EGFR) labelled with ICG, AF750 and IR-Dye800, whereby IR-Dye800 and ICG conjugated cetuximab were both highly specific and sensitive. The validation of BLI and NIRF signals detection is presented in Fig. S2A. We could confirm the expression of EGFR in the MDA-MB-231 orthotopic model with a high specificity. It was easily possible to highlight the optical imaging signals from the background noise (moreover, BLI and NIRF data were compared to the IVIS-Lumina II system, data not shown). With our system, the *in vivo* detectable fluorochromes with PA using this set up were the ICG and the IR-Dye800. AF750 couldn’t be detected *in vivo* 24 H post-injection by PA. In Fig. S1 are shown the *in vitro* spectrophotometric characterizations of the cetuximab constructs acquired using photoacoustics. The major differences that were expected relied on the ability of the PA system to detect these different fluorochromes. The PA absorption peak measured by a Spectro-Acquisitions show slightly shifted peaks in the far red direction as compared to the intrinsic characteristics of the dyes alone. PA absorption peaks measured were respectively 834 nm, 790 nm and 871 nm for the cetuximab-ICG, cetuximab-AF750 and cetuximab-IR-Dyes800.

**Figure 2:**
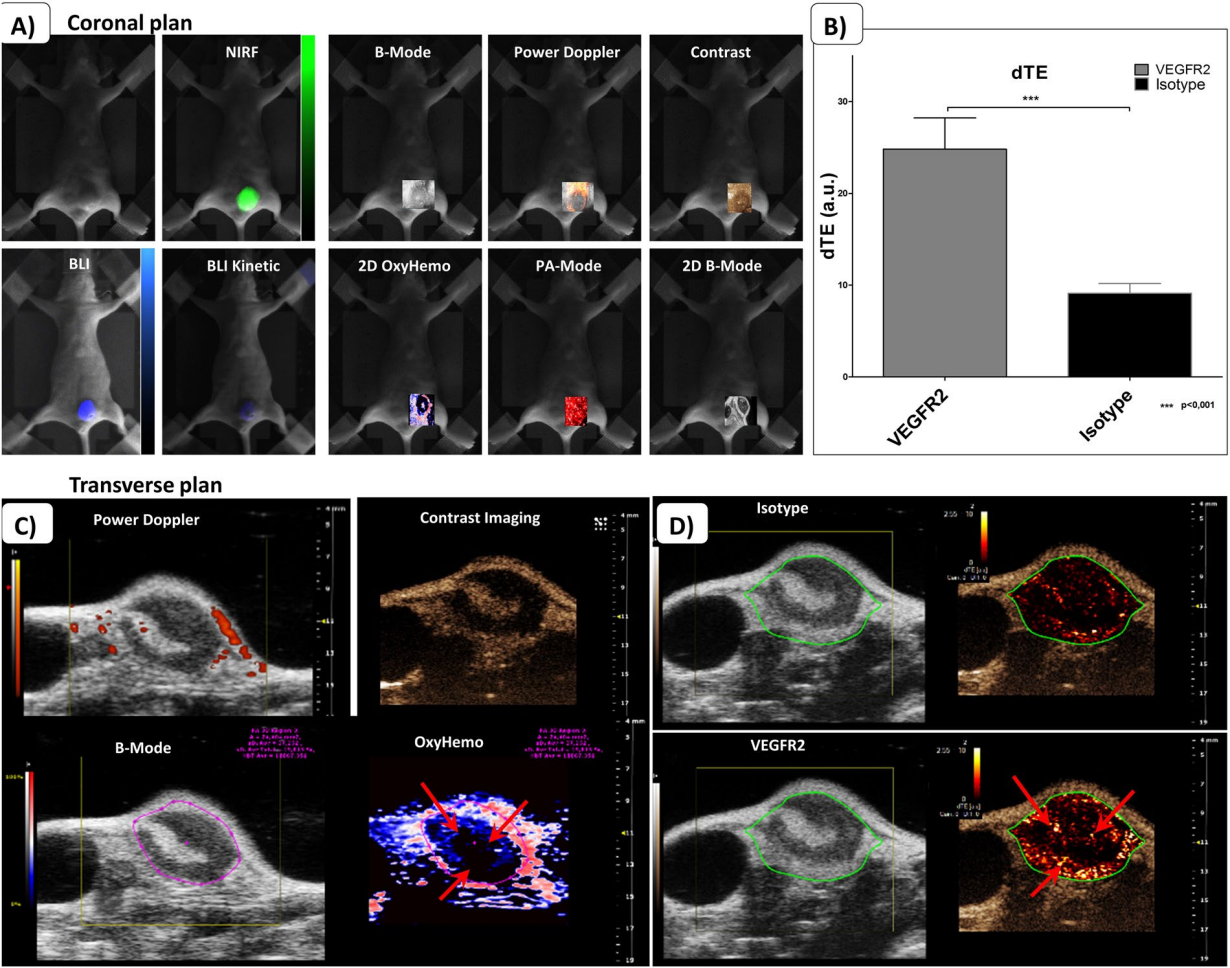
Comparisons of modalities: From biophotonics to ultrasounds and photoacoustics. (**A**) 2 dimensions biophotonics acquisitions (BLI, BLI kinetic and NIRF) are compared to 3D acquisitions from the VEVO system. Here are presented 2D coronal rendering from 3D acquisitions acquired thanks to the stepper motor. Acquisitions for B-Mode, Power Doppler, Contrast, Oxy-Hemo and PA-Mode can easily be merged on black and white image from the camera. (**B**) Assessment of the differential Targeted Enhancement (dTE), following the destruction of both targeted and non-targeted microbubbles. A higher dTE means a greater microbubbles fixation, so an important VEGRF2 expression. (**C**) Examples of comparisons that can be performed on the same slice from the same tumor *in vivo*. In this set of images, we find transverse Power Doppler, Contrast Imaging and Photoacoustic OxyHemo-Mode. Red arrows highlight hypoxic areas corresponding to VEGFR2 expression. (**D**) Corresponding transverse parametric images from ultrasound molecular imaging showing the VEGFR2 expression as compared signals from microbubbles targeted via an isotype antibody.

In Fig. 2A, we compared the data from 2D BLI and NIRF images to 2D images from 3D US and PA acquisitions on the orthotopic MDA-MB-231-Luc2 breast cancer model. We obtained a very sensitive detection of BLI signals from the tumor which allowed us to record BLI kinetics (20 min dynamic acquisitions with 10 sec frames). We could confirm the high expression of EGFR on this MDA-MB-231 model 24 H after the cetuximab-ICG probe, associated to a high proliferation status. We also investigated tumor VEGFR2 expression after injection of targeted microbubbles. Our results demonstrated that there was a significant difference in the dTE from the VEGFR2 targeted microbubbles compared to microbubbles labelled with an isotype control antibody (Fig. 2B). We were able to estimate the VEGFR2 expression inside these tumors using the parametric images from the Contrast-Mode of the US system (Fig. 2C). Moreover, we compared this VEGFR2 expression to Power Doppler and Oxy-Hemo (Fig. 2C) in which we observed that VEGFR2 expression corresponds to highly hypoxic regions. Red arrows indicate hypoxic regions associated to a high VEGFR2 expression (Fig. 2C,D).

Thanks to 3D rendering images from 3D US and PA data, we were able to compare both superficial and deeper data, at any depth level, after a co-registration with BLI and NIRF images. The mouse injected with the cetuximab-ICG presented in Fig. S2A corresponds to the US/PA images presented in Fig. S2B. The representative mouse was imaged by PA before targeted fluorescent probe injection to obtain a background acquisition. 24 H post IV injection of the cetuximab-ICG, the same mouse was imaged again by NIRF, BLI, US, CEUS & PA. PA data were processed in order to unmix the specific signal from the probe. We then compared the 2D EGFR expression and BLI signals not only with the 3D cetuximab-ICG biodistribution measured by PA but also with other US/PA modes available such as 3D B-Mode, Power-Doppler and Oxy-Hemo (Fig. S2B). As the 2D quantification of NIRF is not yet available with our set up, we unfortunately couldn’t compare the 2D EGFR expression levels to other data obtained. However, we could identify 2 regions where the EGFR expression (PA micro-biodistribution of the cetuximab-ICG) corresponds to a relatively high oxygenation status and an important vascularization (power doppler and CEUS) (green Arrows on Fig. S2B).

**Figure 3:**
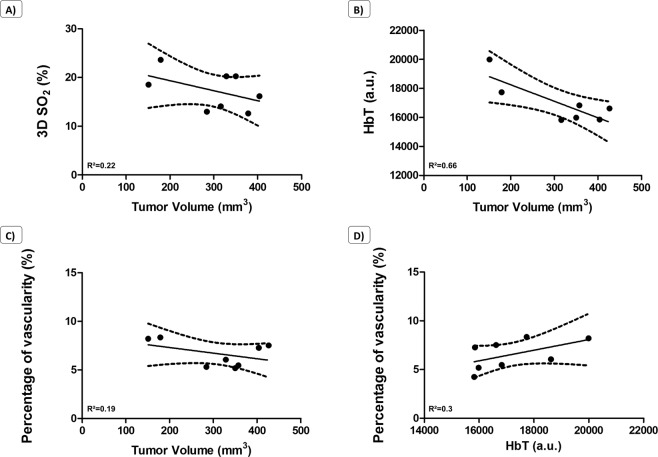
*In vivo* correlation curves from the multimodality approach: US/CEUS/PA. Data are presented with linear regression, 95% confidence-interval estimates and R-squared coefficients (n = 8).

We also could correlate the quantifications from tumor volumes, 3D SO_2_, HbT and percentage of vascularity from Doppler (Fig. 3). On the correlation curves with 95% confidence-interval estimates, we identified trends between these measurements, namely when tumors are growing, the percentage of vascularizaty, the HbT and the 3D SO_2_ are decreasing. The percentage of vascularity seems to be linked to the total content of haemoglobin. When HbT is high, the percentage of vascularity is also high.

In an orthotopic setting using a breast cancer model, BLI allowed the monitoring of tumor proliferation, without quantification, due to the rapid occurrence of hypoxia. NIRF signals were monitored to measure EGFR expression as well as its micro-biodistribution. With US B-Mode, we obtained information on tumors echogenicity and measured tumor volumes. Thanks to Power Doppler, we assessed the percentage of vascularity. PA imaging enabled the assessment of cetuximab micro-biodistribution and the monitoring of hypoxia within tumors. Finally, CEUS imaging provided information on the perfusion status of tumors and the VEGFR2 expression (Figs. S3 and S4).

#### Efficacy assessment of the targeted prodrug

**Figure 4:**
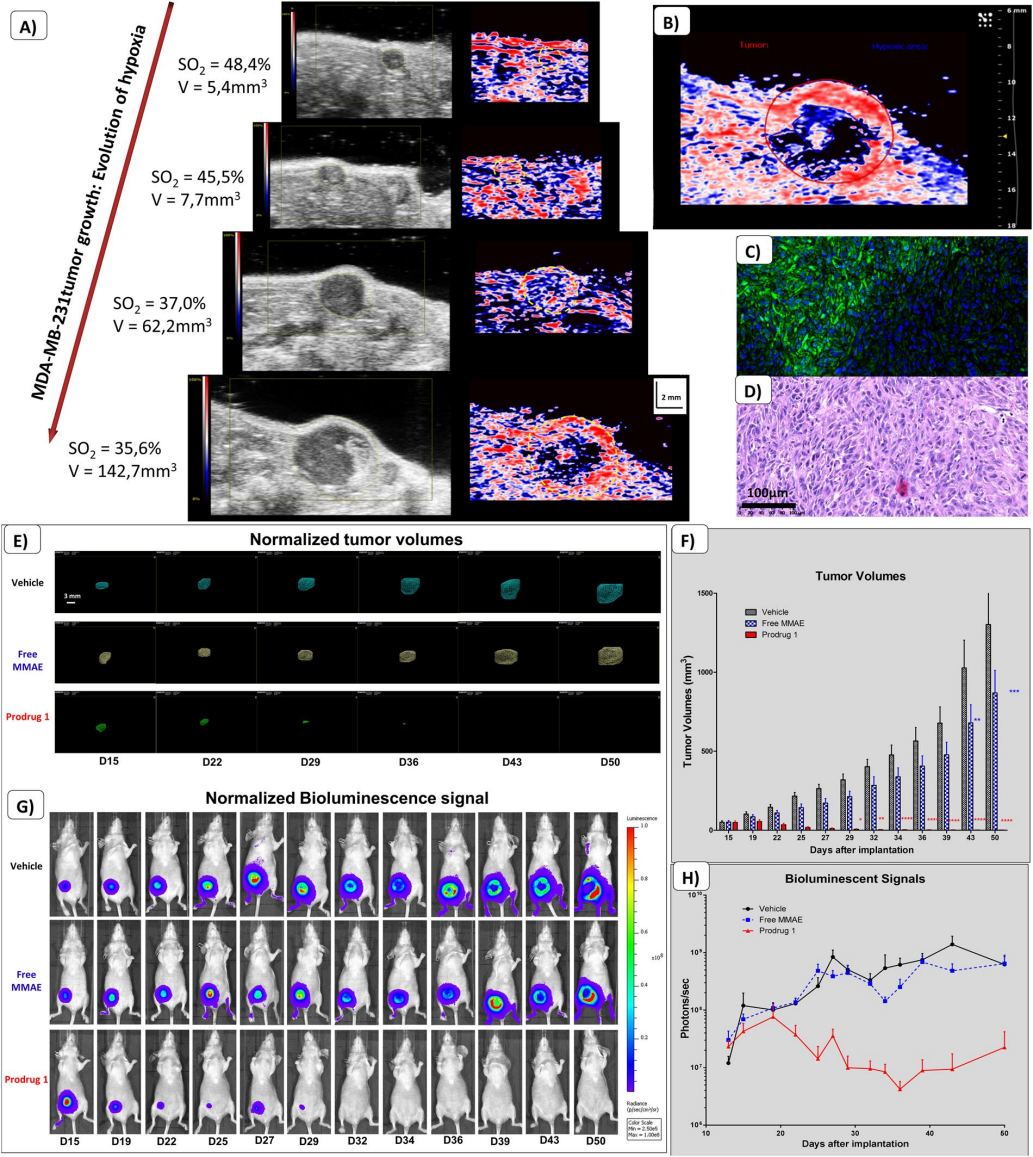
Multimodal imaging of the orthotopic MDA-MB-231 Breast cancer model. (**A**) Representative evolution of tumor growth (from 5.4 mm^3^ to 142.7 mm^3^) with corresponding photoacoustic imaging of hypoxia. (**B**) Delineation of the whole tumor area (red line) compared to the hypoxic area (blue line). (**C**) Pimonidazole immunohistochemistry on MDA-MB-231 tumor sections. Green staining represents pimonidazole adducts (FITC), blue staining represents cells nuclei (DAPI). (**D**) Corresponding hematoxylin/eosin staining. (**E**) Representative evolution of tumor volumes measured by 3D ultrasound imaging. (**F**) Quantifications and comparisons of tumor volumes from the treated group (Prodrug 1) as compared to free MMAE and Vehicle group. Results represent mean ± SEM (n = 6 animals per groups) (previously published data, *Renoux et al*. 2017). (**G**) Representative corresponding bioluminescent signals. (**H**) Quantifications and comparisons of bioluminescent signals from the treated group (Prodrug 1) as compared to free MMAE and Vehicle group. A two-way repeated-measure analysis of variance followed by Bonferroni post-tests was used for the data of over time course. Differences were considered significant at p < 0.05. (*p < 0.05; **p < 0.01; ***p < 0.001; ****p < 0.0001).

Orthotopic breast cancer (MDA-MB-231): A well oxygenated/vascularized peripheral part of tumors presents a high BLI signal compared to the central area (Figs. 4A & 3G). We investigated the appearance of hypoxia over time in MDA-MB-231 tumors by PA imaging. The highlighted hypoxic regions appearing in the core of tumors correlated to a decrease in 3D SO_2_ measurements (a drop from 48% to 36% in a representative tumor; Fig. 4A). We previously set a display threshold on the PA color scale to determine the hypoxic volumes in a reproducible manner (c.f. Material and Methods). This specific threshold has been used to delineate the hypoxic areas on each slice of the 3D acquisition (Fig. 4B). In order to validate our *in vivo* hypoxia measurements, we performed immunohistochemistry using pimonidazole on resected tumor samples. This demonstrated that tissues were already hypoxic, whilst necrosis was not yet present in these untreated tumor samples, based on the H&E staining (Fig. 4C,D).

Following treatment with Prodrug 1, using 3D US imaging we observed a decrease in tumor volumes (Fig. 4E,F) (previously published data, *Renoux et al*. 2017). Moreover, changes in BLI signals from the treated group correlated to the decrease in tumor volumes. However, this trend was not observed in the control groups (Fig. 4G,H). Standard deviations from BLI data compared to US data reflected the inaccuracy of BLI on this particular cancer model. We noticed an initial BLI signal increase followed by a stagnation that could be associated to a delayed peripheral tumor proliferation.

Regarding the antitumor efficacy of the Prodrug 1, we observed significant reductions in tumor volumes measured by 3D US imaging between the treated group as compared to vehicle and free MMAE group. Prodrug 1 was fast acting, whereby tumor volumes started to decrease following only one administration of the compound. At the end of the treatment, complete regression of tumors were observed in 50% of animals, and partial regressions were observed (Fig. 4G).

**Figure 5:**
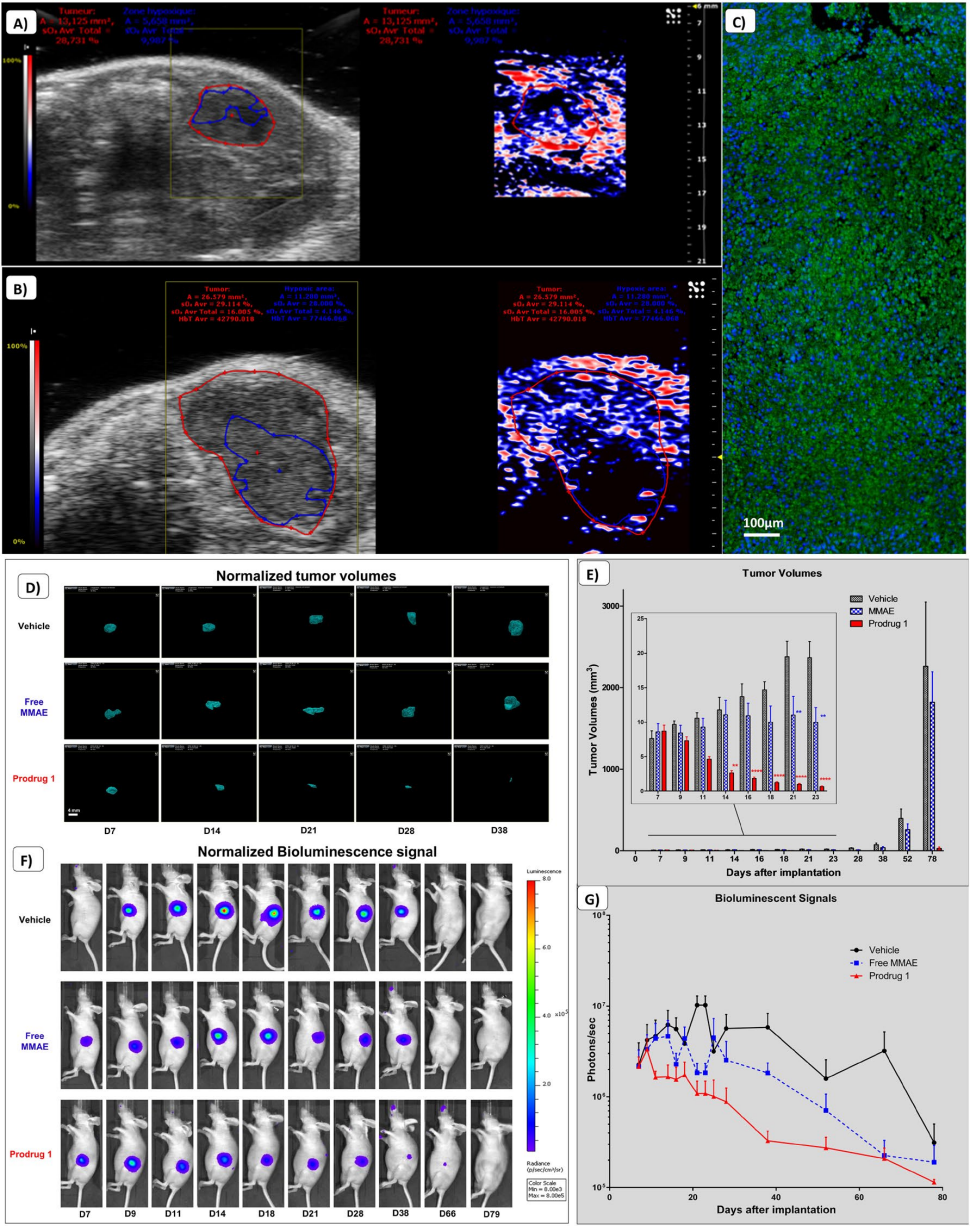
Multimodal imaging of the orthotopic Mia-PaCa2 human pancreatic cancer model. (**A**,**B**) Representative photoacoustic image 17 days and 27 days after engraftment showing hypoxia appearing in the core of the tumor (delineation of the whole tumor area with the red line (from 28% on day 17 to 16% on day 27), compared to the hypoxic area with the blue line (from 9.9% on day 17 to 4.1% on day 27)). (**C**) Pimonidazole immunohistochemistry on Mia-PaCa2 tumor sections. Green staining represents pimonidazole adducts (FITC), blue staining represents cells nuclei (DAPI). (**D**) Representative evolution of tumor volumes measured by 3D ultrasound imaging. (**E**) Quantifications and comparisons of tumor volumes from the treated group (Prodrug 1) as compared to free MMAE and Vehicle group. Results represent mean ± SEM (n = 6 animals per groups) (previously published data, *Renoux et al*. 2017). (**F**) Representative corresponding bioluminescent signals. (**G**) Quantifications and comparisons of bioluminescent signals from the treated group (Prodrug 1) as compared to free MMAE and Vehicle group. A two-way repeated-measure analysis of variance followed by Bonferroni post-tests was used for the data of over time course. Differences were considered significant at p < 0.05. (**p < 0.01; ****p < 0.0001).

Orthotopic pancreatic tumors (Mia-PaCa2): We investigated the development of hypoxia in Mia-PaCa2 pancreatic tumors by PA, whereby we were able to distinguish hypoxic regions in the core of tumors from ~8 days following tumor implantation (Fig. 5A). Overall, decrease in BLI signals due to hypoxia occurred from day 17 post tumor induction, a stage when hypoxia is already significant (SO_2_ = 28% for the whole tumor and SO_2_ = 9.9% for the hypoxic core delineated in blue, in this representative animal). Further, we observed that hypoxic regions encompassed a greater percentage of the whole tumor volume as the tumor grew. We measured, for the same tumor, 27 days after engraftments, a SO_2_ at 16% with a highly hypoxic core (SO_2_ = 4.1%) (Fig. 5B). Immunohistochemistry analysis of pimonidazole revealed hypoxia both at the periphery and in the core of tumors (Fig. 5C).

Regarding the antitumor efficacy of the Prodrug 1, as compared to control groups, we observed a dramatic decrease of tumor volumes after the first administration of the prodrug, and 33% of animals exhibited a complete regression of tumors (previously published data, *Renoux et al*. 2017). Tumor growth was delayed in the free MMAE group, whereas there was a continuous tumor growth for the vehicle group (Fig. 5D,E). BLI signals from animals treated with Prodrug 1 correlated to the decrease in tumor volume by 3D US imaging (Fig. 5F). However, BLI signals were highly disturbed (stagnation then decrease of signals) regarding the control groups from day 20 (Fig. 5F,G). Large variability in the BLI signals compared to tumor volume (3D US) and further discordance between the data from these two modalities also reflected the inaccuracy of using BLI to measure tumor proliferation in this particular cancer model.

### Assessments of tumor hypoxia during treatments as a promising multimodality approach

**Figure 6:**
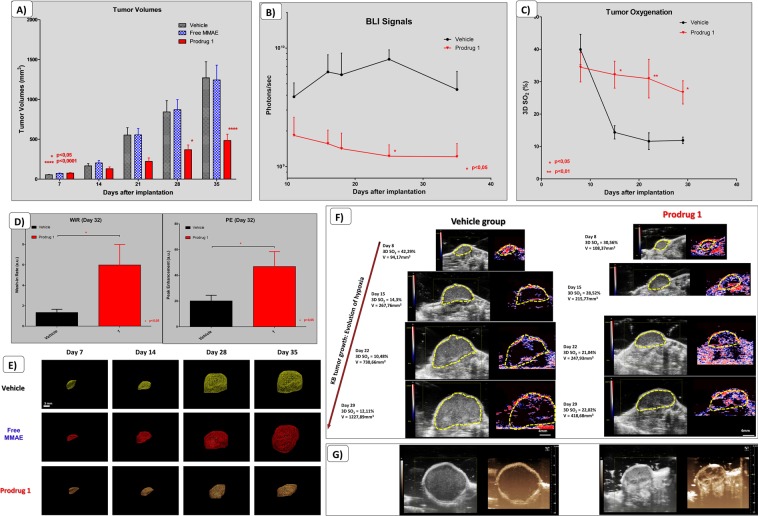
Efficacy assessment on the subcutaneous KB human head & neck cancer model. (**A**) Quantifications and comparisons of tumor volumes from the treated group (Prodrug 1) as compared to free MMAE and Vehicle group (n = 8 animals per groups) (previously published data, *Renoux et al*. 2017). (**B**) Quantifications and comparisons of bioluminescent signals from the treated group (Prodrug 1) as compared to free MMAE and Vehicle group. (**C**) Assessment of tumor oxygenation by 3D photoacoustic imaging in the treated group compared to the vehicle group. A two-way repeated-measure analysis of variance followed by Bonferroni post-tests was used for the data of over time course. Results represent mean ± SEM (n = 4 animals per groups). Differences were considered significant at p < 0.05. (*p < 0.05; **p < 0.01; ****p < 0.0001). (**D**) Quantification and comparison of the Wash in Rate (WiR) and the Peak Enhancement (PE) in the treated group compared to the vehicle group. Statistical analysis was performed with the Student’s unpaired t test (n = 4 animals per group). (**E**) Representative evolution of tumor volumes measured by 3D ultrasound imaging. (**F**) Representative evolution of tumor growth from both treated group and vehicle group, with corresponding photoacoustic imaging of hypoxia. Large hypoxic areas are shown in the vehicle group as compared to the treated group. (**G**) Corresponding Maximum Intensity Persistence (MIP) of non-targeted contrast imaging acquisitions on tumors after 10 seconds following the IV injection of microbubbles.

In the subcutaneous KB head and neck cancer model, selected because of its highly hypoxic potential, we observed a significant difference in the tumor growth for the prodrug treated group compared to control groups (Fig. 6A). Based on the 3D volumes measurements, there was a reduction of the tumor growth without decrease of tumor volumes for the group treated by prodrug 1 (Fig. 6E) (previously published data, *Renoux et al*. 2017). In the vehicle treated group, we observed high variations in BLI signals due to appearance of hypoxia (Fig. 6B), while tumors were still growing. We also assessed tumor oxygenation during the time course of treatments by longitudinal PA imaging. We noticed that oxygenation of tumors was maintained in the prodrug treated group, whereas there was a remarkable decrease in tumor oxygenation observed in the vehicle group. With the aim to confirm our PA results, we performed non-targeted US contrast imaging (Fig. 6D,G). We observed significant differences between the vehicle group and the treated group in both Wash in Rate and Peak enhancement measurements. In an assessment of Maximum Intensity Persistence (MIP) from contrast imaging 10 seconds after IV injections of microbubbles, there was an observed lack of functional vasculature in the vehicle group, explaining the high hypoxia in these tumors (Fig. 6G,F). Conversely, we observed a maintained vascularization associated to a maintained oxygenation in Prodrug 1 treated tumors.

**Figure 7:**
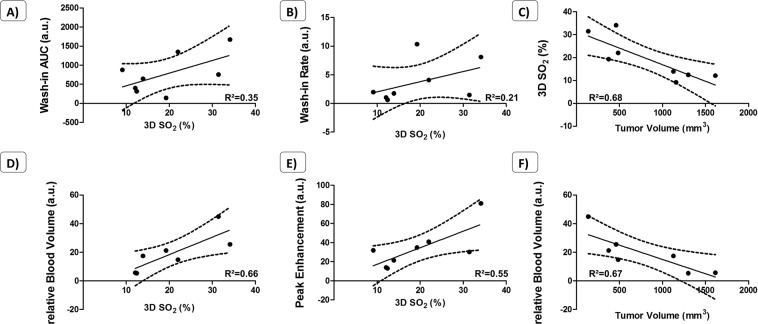
*In vivo* correlation curves from US/PA acquisitions on the KB model. Data are presented with linear regression, 95% confidence-interval estimates and R-squared coefficients (n = 8).

We further investigated the appearance of hypoxia over time in these tumors by PA, where we observed development of hypoxic regions in the tumors from the vehicle group associated to a high decrease of 3D SO_2_ (from 42% at day 8 to 12% at day 29 in this representative animal, Fig. 6F). In comparison, we also observed hypoxic regions in the Prodrug 1 treated group. However, these regions compromised a smaller percentage of the total tumor volume, and there was a smaller decrease in 3D SO_2_ (from 30% to 22%). When comparing CEUS data, tumor volumes and 3D SO_2_, we could highlight trends from the correlation curves with 95% confidence-interval estimates. Indeed, the more hypoxic the tumors are, the less vascularized they are (Fig. 7). Bigger tumors also present a lower relative Blood Volume. Parameters extracted from non-targeted CEUS are lower in poorly oxygenated tumor tissues (e.g. WiAUC, WiR, rBV & PE). Moreover, the bigger the tumors are, the more hypoxic they are, and inversely.

## Discussion

### General remarks

We described for the first time a multimodal platform suitable for *in vivo* PA, US, BLI and NIRF imaging, within the same light enclosure from the Vevo LAZR system (FUJIFILM Visualsonics Inc.). We selected a suitable camera to be implemented in this platform as the whole set up is not commercially available. This homemade build platform, which combines highly sensitive 2-dimensional whole body imaging techniques (e.g. biophotonics) with high resolution images from 3-dimensional cross-section imaging modalities (e.g. ultrasounds and photoacoustics), is suitable for high throughput imaging series, thanks to the single animal anesthesia procedure required for both steps of biophotonics and ultrasound based imaging protocols.

The fact that these modalities are either real-time or last few minutes enables the very precise monitoring of each lesion. Depending on the data obtained, this approach allows the scientists to make decisions regarding the becoming of animals (e.g. more precise data needed associated to deeper analysis, administration of new treatments, longer monitoring, or sacrifice) without changing the scientific outcome. One of the challenges was also to speed up the imaging session, minimising animal distress and improve welfare. It eases the workflow for researchers as the modalities implemented are very fast, so it does not take a long time to acquire basic data such as BLI, NIRF or US. With respect to high resolution PA imaging or CEUS acquistions, several minutes are necessary to obtain the required information with the desired quality. Moreover, scientists can select which modality is the most relevant for ongoing studies.

This set up avoids the use of different beds or masks, shortening the length of animals’ anaesthesia and decreasing the total amount of time for the data collection. It is thus of great interest regarding the animal welfare and strongly supports the 3Rs in animal science.

The significant advantages of the proposed approach are the direct comparison of anatomical and functional data. The modalities implemented allow the operator to precisely monitor the lesions with the dedicated modality. In addition, the high throughput of acquisitions and data acquired provide relevant information promptly. The proposed approach facilitates the detection and the follow up of the primary tumors’ exact location, as well as the location of tumor margins and metastasis with the different available modalities (for example the early detection of metastasis with BLI or NIRF with a US or PA follow up to determine its growth, oxygenation status or endothelial receptors expression). The perfect merging of acquired images (e.g. epi-illumination NIRF & BLI images recorded on top of the animal, and US/PA transducer applied on top of the animal body) is decisive.

The complementarity of such a combination allowed to understand how correlated the vascularization parameters are with the SO_2_ and how it evolves alongside tumor growth. Tumors oxygenation appeared closely linked to the tumor vascularization status. Parameters extracted from non-targeted CEUS such as rBV, WiR, WiAUC and PE are either influencing or influenced the tumor SO_2_. However, for now, we couldn’t identify which phenomenon is appearing first? Is it the lack of functional vasculature that will provoke tumor hypoxia, or it is the tissue hypoxia that will switch the vasculature into a disorganized network. We are convinced that only multimodal approaches confronting could help giving answers and strategies to design new drugs, especially by studying the *in vivo* tumor vasculature’s destruction or normalization. Despite this unique confrontation of results, much more interesting studies could be designed in order to understand mechanisms and identify new hallmarks of cancer.

Each imaging modality has its own advantage and limitations, yet together provide important complementary diagnostic information^23^. As such, within the preclinical molecular imaging field, numerous studies implementing multimodal imaging have already been performed. A usual multimodality approach often combines BLI and NIRF as these imaging requires the same experimental environment and camera^24^. Combinations of US with NIRF^25,26^, US/PA with NIRF^6,27–29^, or of US with MRI and BLI^30^, have also been investigated. Some combinations with radioactive modalities are also described in the literature, such as PET with NIRF^31^, PET/CT with BLI^32^ or SPECT/CT with BLI & NIRF^33,34^. However, as radiation from both CT based anatomical imaging and radioisotopes can influence organ and cell biology leading to biased experimental data^7^, we developed a non radiation-based imaging platform.

### Bio-photonic imaging

The main advantage of biophotonics is in the early and sensitive detection of tumors and accurate localization of metastases. Differential information provided by BLI (e.g. tumor proliferation, receptors expression with genetically engineered animals) and NIRF (e.g. biomarker expression), is of interest particularly when one of these modalities reaches its limitations. Biophotonics procedures are fast and easy to use, when carefully taking into account the well-known limits. However, a parameter that must be considered with optical imaging is the quantification of signals. Indeed, the disadvantage of optical imaging is the penetration and scattering of light in the tissue^35^, where strong photon absorption and scattering limits this modality to small animal or superficial clinical applications^7^. BLI acquisition signals are often quantified in counts^36^, however regarding our platform, as we did not calibrate signals with a dedicated light source, we could not quantify the emitted signals yet.

The assessment of signals from tumors presenting a certain degree of hypoxia showed significant reductions regarding the quantification (IVIS-Lumina II measurements). *Khalil et al*. also showed that both appearance of hypoxia and changes in pH influence BLI signals^37^. Since the bioluminescence reaction is dependent upon the presence of ATP and O_2_, it cannot be used as a relevant proliferation biomarker when a tumor becomes hypoxic. This phenomenon is particularly evident in the vehicle groups for pancreatic and head and neck tumors which exhibited progressive decrease of BLI while tumors volumes were still growing (Figs. 5D,E and 6B). For breast tumors, disturbances in BLI were more complex with a two-phase process associated to a delayed peripheral tumor proliferation (Fig. 4G,H). BLI seemed to be promising to follow orthotopic tumor growth, as tumors were deeper in animal bodies. However, 3D US imaging highlighted the lack of relevance and reliability of BLI quantification in these particular hypoxic cancer models.

Another limitation of BLI is the strong absorption of photons by crossed tissues making the quantification and interpretation challenging with this planar modality^38^. Nonetheless, BLI remains a unique modality to follow both early stages of proliferation and early metastasis. This can allow allocations into experimental groups of animals with equivalent tumors stages, for more robust experimental design. Regarding BLI kinetics measurement, it was used to determine which lesion was the most active (especially when implementing metastasis models), but was also used to determine when is the best time window to perform BLI acquisitions after the IP administration of luciferin to animals (determining when the plateau phase is reached for each particular tumor model).

For fluorescence investigations, we tested different administered doses to be detected in animals, and the ORCA camera was as efficient as the IVIS regarding the signal to noise ratio. But unfortunately, no minimal concentrations were derived using the platform with those different animal models. The cetuximab-ICG construct allowed the clear delineation of tumors with NIRF signals, thus confirming the EGFR expression in this model. However, the labelling stability was shorter, and the background noise became more and more important as compared with the 2 other fluorochromes when used more than 20 days after the labelling process (data not shown). Indeed, after that time we could notice non-negligible signals coming from the liver, and viscera for some animals (the elimination of ICG dye is mainly performed by intestinal and biliary routes after hepatic filtration).

One of the NIRF imaging limitation is due to the tissue absorbers (i.e. oxy-haemoglobin, deoxy-haemoglobin, water, lipids or melanin)^39^. For orthotopic examinations, the auto-fluorescence from skin and particularly the food present in viscera has to be considered before planning an experimental scheme. To avoid this, a specific non-fluorescent food may be given to animals 3 days prior to imaging sessions until the end of fluorescent examinations. Further, in this work, we use NIRF dyes designed in absorbing around 750 nm, thus avoiding most of the auto-fluorescence of skin and tissues.

In the past decade, lots of efforts have been made to improve the detection of deep foci in animals using NIRF, in particular by using different approaches regarding the illumination process of tissues (e.g. epi-illumination and trans-illumination). Epi-illumination technique consists in a surface illumination of tissues and the collection of the emitted light with both the source of light and the camera located on the same side (also called reflectance). As photons are able to scatter in tissues, the light reaching the surface can propagate few millimetres and excite fluorochromes under the surface. The emitted light by the fluorochromes can be collected by the camera mounted with appropriate filters. Epi-illumination is a simple technique routinely implemented for screening with acquisitions only lasting seconds. This very popular technique enabled important breakthrough regarding fluorescence molecular imaging. However, this approach lacks the spatial depth discrimination. Although the detected fluorescence intensity linearly depends on the concentration of fluorochrome or the amount of fluorochrome present in a foci (a tumor for example), it exists a non-linear relation with the depth of a foci, its optical properties and surrounding tissues. Two tumors with the same concentration of fluorochrome will not have the same fluorescence intensities using epi-illumination if they are located at different depth or if their vascularization is different. Trans-illumination is a planar imaging technique, transmitting the exciting light through the tissue: the source of light and the camera are disposed on each side of the tissue. It is of course possible to collect the emitted fluorescence but also to determine the attenuation of light from the tissue by collecting the source light. Trans-illumination has the advantage of limiting autofluorescence because the surface where the signal is detected is not illuminated by the exciting light. Moreover, by exciting the entire volume of interest with a crossing light, it would be able to image deeper foci than epi-illumination. This planar imaging modality is less used in than epi-illumination, but it is used in recent systems of fluorescence molecular tomography^40^.

The spectral shift in biophotonics is a well observed and studied phenomenon that happens at longer wavelengths due to the tissue absorption, the thickness and the nature of tissues crossed. For example, in BLI, the major part of photons collected by the cameras belongs to the end of the luciferin spectrum (600–650 nm). However, when using interferential filters with narrow bandwidths, the spectral shift observed can be correlated to the depth of the foci. It then enables the allocation of signals to different foci depths and locations. For now, the only method considered to assess the absorption of photons by tissues present between the skin surface and the source of photons is this spectral analysis^38,41^. This technique is mainly used to determine the depth of a foci depending on the nature and depth of tissue crossed, but not as a routinely used^42^. We can cite a work from *Dehghani et al*. where the team worked on a quantitavive bioluminescence tomography approach using spectral derivative data^43^. The scattering of photons is also a factor contributing to the degradation of performances in BLI and NIRF. Tissue scattering decreases the spatial resolution of images acquired^44^. Knowing how challenging *in vivo* optical imaging is, we noticed that numerous research teams worked on different approaches using smart image processing or computer-aided analysis tools, enabling new parameter measurements that could not be studied and quantified before^45–48^.

### Ultrasound imaging

Ultrasound imaging has the capability to both determine tumor volume, and further assess angiogenesis. Power-Doppler provides information on blood flow in vessels bigger than 100 µm in diameter, whereas non-targeted CEUS imaging using MBs can detect flow parameters from smaller vessels (as the time MBs size is less than 5 µm in diameter)^49,50^. The technology of US is very attractive for onco-pharmacology studies because it is absolutely inert regarding the tumor growth and it does not implement ionizing radiation^51^. 3D ultrasound measurements are much more accurate, relevant and reproducible, regarding determination of volumes for both superficial and internal tumors, as compared to calliper measurement. The disadvantage of calliper measurements for subcutaneous tumor xenografts is both the lack of applicability for metastasis assessment^52^, and issues due to irregular tumor shapes, as formulas have to be applied to obtain tumor volumes. In these studies, we could measure the tumor volumes in an accurate way, and we highlighted the differences in the tumor vasculature with CEUS using MBs between control and Prodrug treated groups.

### Photoacoustic imaging

The strength of the PA system is the pulsed laser enabling deeper light penetration through tissues as compared to conventional filtered white light. PA imaging is a hybrid biomedical imaging method presenting advantages from optical imaging and US so that it allows measurements of physiological as well as structural information^53^. This technique is constantly and rapidly advancing toward higher sensitivity and resolution^15^. PA imaging provides unique assessment in the evolution of hypoxia in tumors and has the potential to account for the limitations of BLI in models of cancer therapy^54^. PA imaging coupled with US would be of great interest on the assessment of therapies targeting hypoxia into tumors, or of prodrugs becoming active only in the vicinity of tumor hypoxic cores.

We are convinced that every modality implemented in this platform should be used together in order to overcome limitations from others. It could be use determine which kind of hypoxia the tumors are presenting (e.g. chronic, acute or anaemic hypoxia). Chronic hypoxia, is the result of an increase in the distance between blood vessels and tissues, no longer allowing a good diffusion of oxygen. This phenomenon occurs when tumors grow rapidly, and the need for O_2_ is too important. Tumor cells located at about 80 μm of the blood vessels have their O_2_ intake decreased, and the more this distance increases, the more hypoxic it will be. In addition, the circulation directions in the micro-vessel network associated with a less functional endothelium exacerbate this process^55^. Anaemic hypoxia results from a reduction in O_2_ transport capacity. Different studies indicate that oxygen carrying capacity for tumors is reduced. A haemoglobin level below the normal threshold in an subject could contribute to tumor hypoxia. This effect is even more intense when anaemic hypoxia is associated with a decreased tumor perfusion rate^55,56^. Acute hypoxia is caused by insufficient blood flow to the tissues. Due to rapid proliferation and disturbed neo-angiogenesis, tumor microvasculature becomes structurally and functionally abnormal. We can find organization of the vascular network abnormalities, or defects in the regularity of endothelium fenestrations, dilatations or elongated and tortuous vessels. These combined factors cause a decrease in intravascular pressure, which prevents the blood from penetrating properly inside the tumor^55^. Determining the type of hypoxia associated with vascularization parameters and receptors expression could influence the decisions taken regarding the family of anticancer drugs given for a particular tumor type (e.g. antiangiogenic, alkylating agents, antimetabolites, purine or pyrimidine antagonists, alkaloids, antibiotics, hormonal agent…).

An important limitation of PA imaging is the current lack of dedicated and commercially available contrast agents. Many research teams are currently working on dedicated PA fluorochromes with better detection properties. Certain fluorochromes are identified as potentially promising PA contrast agents, yet the limited accumulation of the fluorochromes in the target tissue challenges the limited sensitivity of PA compared to NIRF imaging. Certain fluorophores for PA imaging also have a propensity to bleach or degrade when irradiated by the PA laser pulses and have a relatively small absorption^57^.

The micro-biodistribution of contrast agents is an interesting field of application that can be achieved with PA imaging. Some of our preliminary results showed a strong potential of PA imaging in detecting the micro-biodistribution of labelled monoclonal antibodies. This was demonstrated with the use of an ICG-labelled cetuximab antibody using Power-Doppler, Oxy-Hemo and Contrast acquisitions (Fig. S2). However, these assessments remained challenging due to the contrast agent implemented (particularly when using tracing doses of labelled antibodies). The different cetuximab constructs were used to test their potential as Photoacoustic contrast agents (precisely multimodal agents, as they were already well characterized and efficient for NIRF imaging, but not yet as PA contrast agents). ICG was detectable *in vivo* post 1 minute and 24 H post IV injection of the labelled cetuximab-ICG, but unfortunately, the cetuximab-AF750 was not. The development of cetuximab labelled with IRDye800, a more promising fluorochrome for PA investigations, may allow for improved assessment of EGFR micro-biodistribution.

### Tumor models

Among available tumor cell lines, the Mia-PaCa2 cell line (human ductal pancreatic adenocarcinoma), representative for 80% of pancreatic cancer appeared suitable for such investigations^58^. MDA-MB-231 cell line (human breast adenocarcinoma), being a triple negative model, is associated with a poor prognosis for patients. The three cancer models were quite relevant for these preclinical investigations as the hypoxic status of each tumor type was characterized by PA imaging. Moreover, the characterization of hypoxia on the Mia-PaCa2 and MDA-MB-231 model by pimonidazole immunohistochemistry staining confirmed our Oxy-Hemo investigations^59^.

### Prodrug efficacy

The present study demonstrates that this prodrug is significantly effective on three different hypoxic models of human cancer as compared to the group treated by the parental cytotoxic agent (MMAE) and the vehicle group. This particular prodrug offers interesting perspectives for treating tumors with chemotherapy while sparing healthy tissue^60^. Vectorization of anticancer agents seems to be a very promising concept that could improve significantly the effectiveness of anticancer chemotherapy in a context of translational research. Moreover, considering the crucial effect of hypoxia upon chemosensitivity towards antitumor drugs, the use of characterized hypoxic tumor models should allow better predictions for translation in to clinical research. PA imaging co-registered with high resolution US measurements ensures relevant assessment of therapeutic effects in hypoxic models of human tumors.

The main advantages here of using 3D US measures, compared to callipers measures, are the accuracy, the reproducibility of the data and its need for the monitoring the orthotopic pancreatic tumor model. 3D US measurements, even performed by different operators are very accurate especially for such small tumors (it was not the case in these studies; every acquisitions have been quantified by the same person in order to avoid any operator-dependent variations). Some of our previous results informed about the extreme efficiency of the prodrug associated to an extreme decrease of tumor volumes. To be able to accurately measure the decrease in tumor size, it was clear that the only accurate way to monitor the tumor volumes was the 3D US measure. Indeed, after the treatments in the breast and pancreatic models, we couldn’t see neither palpate the tumors. It was sometimes difficult to find the tumors with US, even in 20 g nude mice, with a very thin skin. As we also wanted to get information on the echogenicity of the tumors, 3D US scans were always performed, allowing the measurement of tumor volumes (we also wanted to investigate whether the observed echogenicity patterns of certain tumors could be correlated to the SO_2_, or the receptors expression).

### Future improvements

The current major limitation of biophotonics in our platform is the quantification of signals. Regarding BLI signal quantification, we will need to calibrate the signals with a source of light associated to image post processing. An optimized design of the actual set-up with a dedicated application will achieve the use of a co-axial circular illumination device, which already improved the homogeneity of illumination allowing a relative quantification. We would also use dedicated camera lenses, with different focal length in order to be closer to the tumor/organ of interest to gain a greater image resolution. Another improvement will focus on the use of the VEVO LAZR pulsed laser, allowing a mono-wavelength illumination of the animal, thus avoiding the use of excitation filters. Because both the camera and the PA/US imaging system can be triggered on the respiratory gating or on the ECG (thanks to the ECG analysis from the pads on the VEVO LAZR imaging station), a motion correction can be carried out and data from foci located near the heart or the thoracic cage can be documented accurately. In further experiments, we also would implement the Black Hole Quencher 3 (BHQ3) which is a commercially available fluorochrome with a potential better yield than AF750 for PA imaging^27^. As external landmarks were used to merge images from the US/PA onto images from biophotonics through digital image manipulation techniques, we would like to develop an automated fusion protocol to merge images automatically.

Using US and PA imaging, it would be possible to monitor early primary tumor growth as well as small metastases due to concomitant detection performed by optical imaging. This approach could allow us to study the biology of metastasis formation and progression. A primary investigation using this particular development allowed to detect and monitor both the tumor growth and the appearance of hypoxia with US and PA. Thanks to the high sensitivity of BLI we were able to follow the appearance of metastasis from the primary tumors (data not shown). Once detected with BLI or NIRF, tumors could be investigated by US (echogenicity, volume), informing on the vascularity (Power Doppler) or the perfusion status (MBs), moreover on the oxygenation status (PA). As BLI kinetics can be recorded with the CMOS camera, it would be of great interest to assess the time shift of BLI signals between the primary tumor and metastases after the administration of luciferin (Fig. S5).

An interesting application of this platform for oncology research would be the *ex vivo* assessment of excised tissues. A work from *Sun et al*. already described the *ex vivo* examination by NIRF/US/PA^61^. So this multimodality approach could be useful for direct examinations on excised tumors or tissues such as sentinel lymph nodes aiming to detect tumor margins or quantify the tumor invasion in the clinical practice.

This multimodal approach would also be useful to perform mechanistic studies, particularly with transgenic animals expressing specific reporter genes for the study of both tumor development and expression of particular proteins^62^. With the use of genetically engineered mice expressing luciferase under the control of specific promoters, this platform could also be of great interest not only in cancer research, but also in the inflammation or infection fields.

## Conclusion

Our results confirmed the ability of this new embedded platform to detect BLI and NIRF signals in first step, and then acquire US and PA data in a second step. It opens new perspectives for biological applications considering the connections between hypoxia and neoangiogenesis. This multimodal approach allows unique confrontation of information provided by PA/US (3D or 2D B-Mode, Doppler, Contrast, PA-Modes, PA spectral unmixing) with a variety of available NIRF biomarkers (integrins, carbonic anhydrase, proteases activatable probes…), as an alternative of radiation-based examinations. Moreover, hypoxic status, tumor metabolism, apoptosis, gene expression and parameters linked to the tumor microenvironment can be monitored longitudinally following a defined procedure depending on the purpose of the study. This platform is also of great interest regarding the development of dedicated multimodal contrast agents (e.g. activatable probes, delivery of PA contrast agents via encapsulation in microbubbles or nanodroplets, …).

For *in vivo* imaging evaluation of the tumor growth and treatment efficacy follow up, it is important to elaborate multimodality studies. Monitoring the perfusion status also highlights effects of the prodrug on the highly hypoxic KB Head and Neck cancer model. This interesting approach therefore brings new hopes for the assessment of anticancer therapies on hypoxic tumors and their microenvironment, especially on well-established hypoxic tumor models known to be chemoresistant and radioresistant. Considering the pivotal effect of hypoxia on the tumor cells sensitivity against anticancer treatments, the use of hypoxic tumor models should allow better predictions for clinical translational research. The proposed imaging technique combination is unique knowing the possible confrontation of information, overcoming other modalities limitations. Further experiments could assess the efficacy of this prodrug combined with radiotherapy. A new version of the prodrug, allowing the release of 2 MMAE molecules will also be tested.

Preclinical evaluation by multimodal imaging of new targeted therapies or prodrugs exhibiting improved half-life is of interest to enhance the efficiency of the drug delivery system. Identifying and characterizing the *in vivo* pharmacologic activity of new anticancer agents, moreover, enabling the use of a lower number of animals is mandatory for the future of small animal molecular imaging. Preclinical imaging is internationally recognized as strategic for innovation as well as for pharmaceutical development, and it will be soon possible to expand the scope of data obtained from such a platform, enabling the identification of specific targets for patient-tailored therapies.

## Supplementary information

Supplementary information
