# Supplementary material for: Development of an embedded multimodality imaging platform for onco-pharmacology using a smart anticancer prodrug as an example
Source: Sci Rep. 2020 Feb 14;10:2661. doi: 10.1038/s41598-020-59561-8 (PMC7021674; doi:10.1038/s41598-020-59561-8)
Supplement: Supplementary file 1 — Supplementary information [file 41598_2020_59561_MOESM1_ESM.pdf]

Development of an embedded multimodality imaging platform for onco-pharmacology using a smart anticancer prodrug as an example

Florian Raes[1,2*], Serigne Moussa Badiane[3], Brigitte Renoux[4], Sébastien Papot[4], Stéphanie Lerondel[2], Alain Le Pape[2]

[1] Institute for Cancer Research, ICR, Sutton, London, UNITED KINGDOM
[2] Centre for Small Animal Imaging, CIPA, Phenomin-TAAM CNRS UPS44, Orléans, FRANCE
[3] UFR des Sciences de la Santé, Université Gaston Berger, Saint-Louis, SENEGAL
[4] IC2MP, Chemistry Institute of Poitiers: Materials and Natural Ressources; Programmed Molecular Systems Team, UMR-CNRS 7285, Poitiers, FRANCE

***Corresponding author:**
Florian RAES, PharmD, PhD
florian.raes@icr.ac.uk

Serigne Moussa BADIANE, MD, PhD
semobadiane@yahoo.fr

Brigitte RENOUX, PhD
brigitte.renoux@univ-poitiers.fr

Sebastien PAPOT, PhD
sebastien.papot@univ-poitiers.fr

Stéphanie LERONDEL, PhD
stephanie.lerondel@cnrs-orleans.fr

Alain LE PAPE, PharmD, PhD
alain.lepape@univ-tours.fr

FIGURES CAPTIONS

Fig S1. *In vitro* spectrophotometric characterizations of the cetuximab constructs acquired using PA. PA absorption peaks measured were respectively 834nm, 790nm and 871nm for the cetuximab-ICG, cetuximab-AF750 and cetuximab-IR-Dyes800.

Fig S2. Validation of the cetuximab labeling by fluorochromes. (A) Validation of the cetuximab antibody labeled with AF750, IR-Dye800 and ICG. BLI and NIRF acquisitions performed with the ORCA camera with Black and White (B&W) reference. (B) Validation of the cetuximab-ICG labelled antibody by PA imaging, and comparisons with the data provided by US and PA. Identification of two regions (green Arrows) where the EGFR expression (PA micro-biodistribution of the cetuximab-ICG) corresponds to a relatively high oxygenation status and an important vascularization (power doppler and CEUS) (the mouse injected with the cetuximab-ICG presented in Fig S2A corresponds to the US/PA images presented in Fig S2B).

Fig S3. Implementation of the imaging platform. A typical example of what can be assessed a given animal longitudinally with each imaging modality implemented with the imaging platform.

Fig S4. Assessment of different physio-pathological parameters in a mouse. (A) 2D Bioluminescence imaging. (B) 2D Fluorescence imaging. (C), (D), (E) & (F) Comparison of 3D rendering from the VEVO system. (G) Corresponding photoacoustic and ultrasound 2D acquisitions allowing the comparison of each acquisition from the different modalities on the same slice of the tumors.

Fig S5. BLI kinetics. BLI kinetics from a spontaneous metastatic model of human lung tumor showing the shift in BLI signals appearance from metastases as compared to the primary orthotopic lung tumor with time (The primary lung tumor being highly hypoxic, no BLI signals from this primary tumor was detectable).

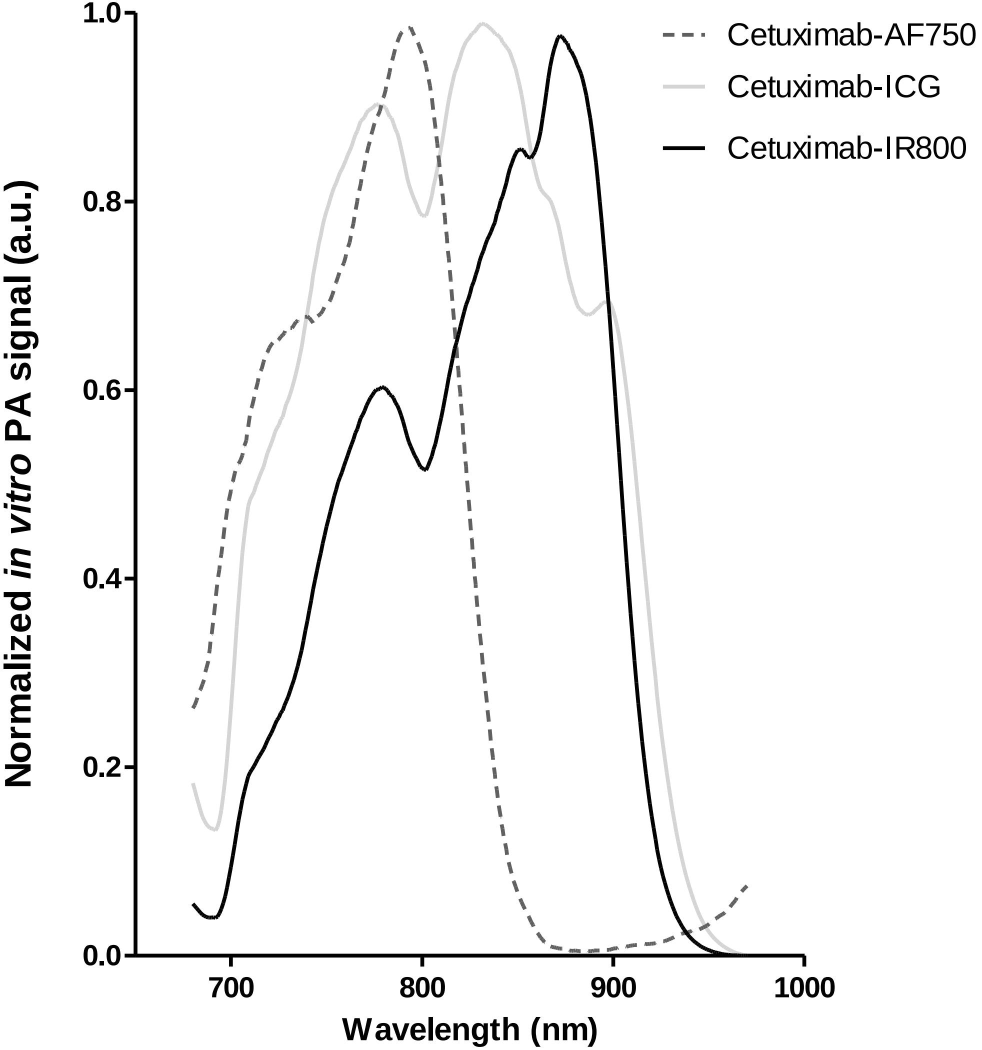

Cetuximab-AF750
Cetuximab-ICG
Cetuximab-IR800
Normalized *in vitro* PA signal (a.u.)
1.0
0.8
0.6
0.4
0.2
0.0
700
800
900
1000
Wavelength (nm)

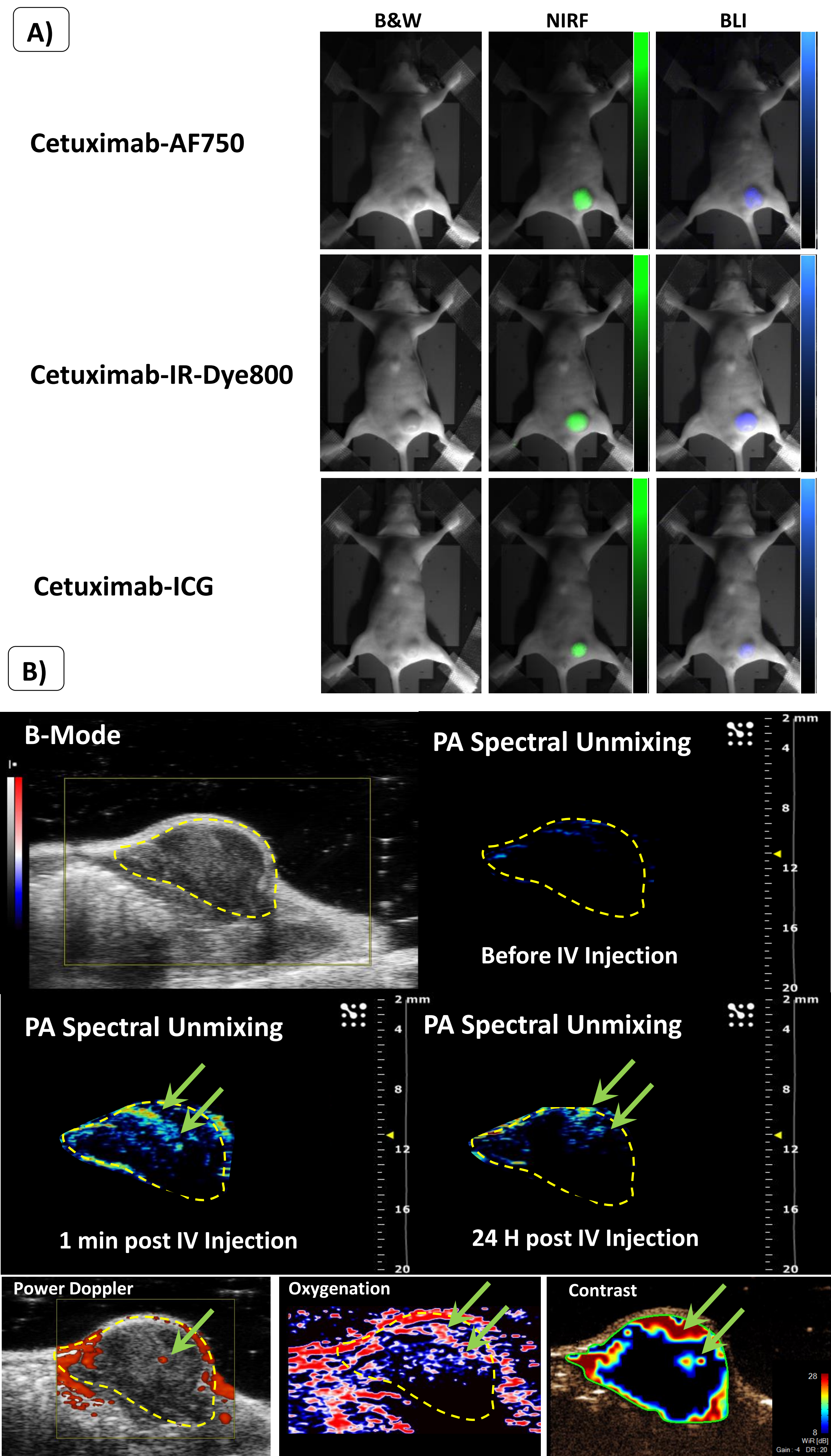
A)
B&W
NIRF
BLI
Cetuximab-AF750
Cetuximab-IR-Dye800
Cetuximab-ICG
B)
B-Mode
PA Spectral Unmixing
Before IV Injection
PA Spectral Unmixing
1 min post IV Injection
PA Spectral Unmixing
24 H post IV Injection
2 mm
4
8
12
16
20
Power Doppler
Oxygenation
Contrast

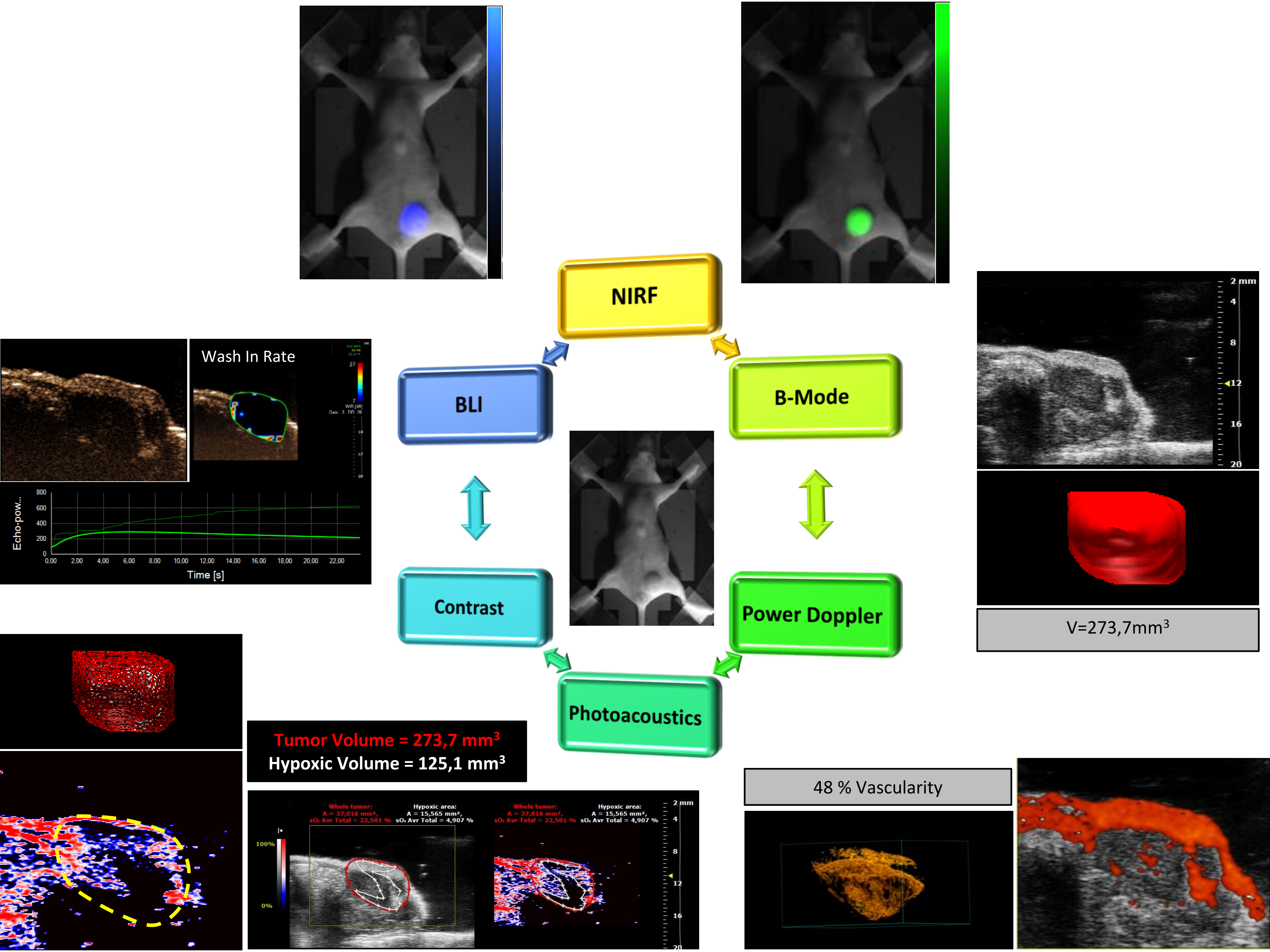

NIRF
BLI
B-Mode
Contrast
Power Doppler
Photoacoustics
Wash In Rate
Echo-pow...
Time [s]
V=273,7mm3
Tumor Volume = 273,7 mm3
Hypoxic Volume = 125,1 mm3
48 % Vascularity
Whole tumor:
Hypoxic area:
100%
0%
2 mm

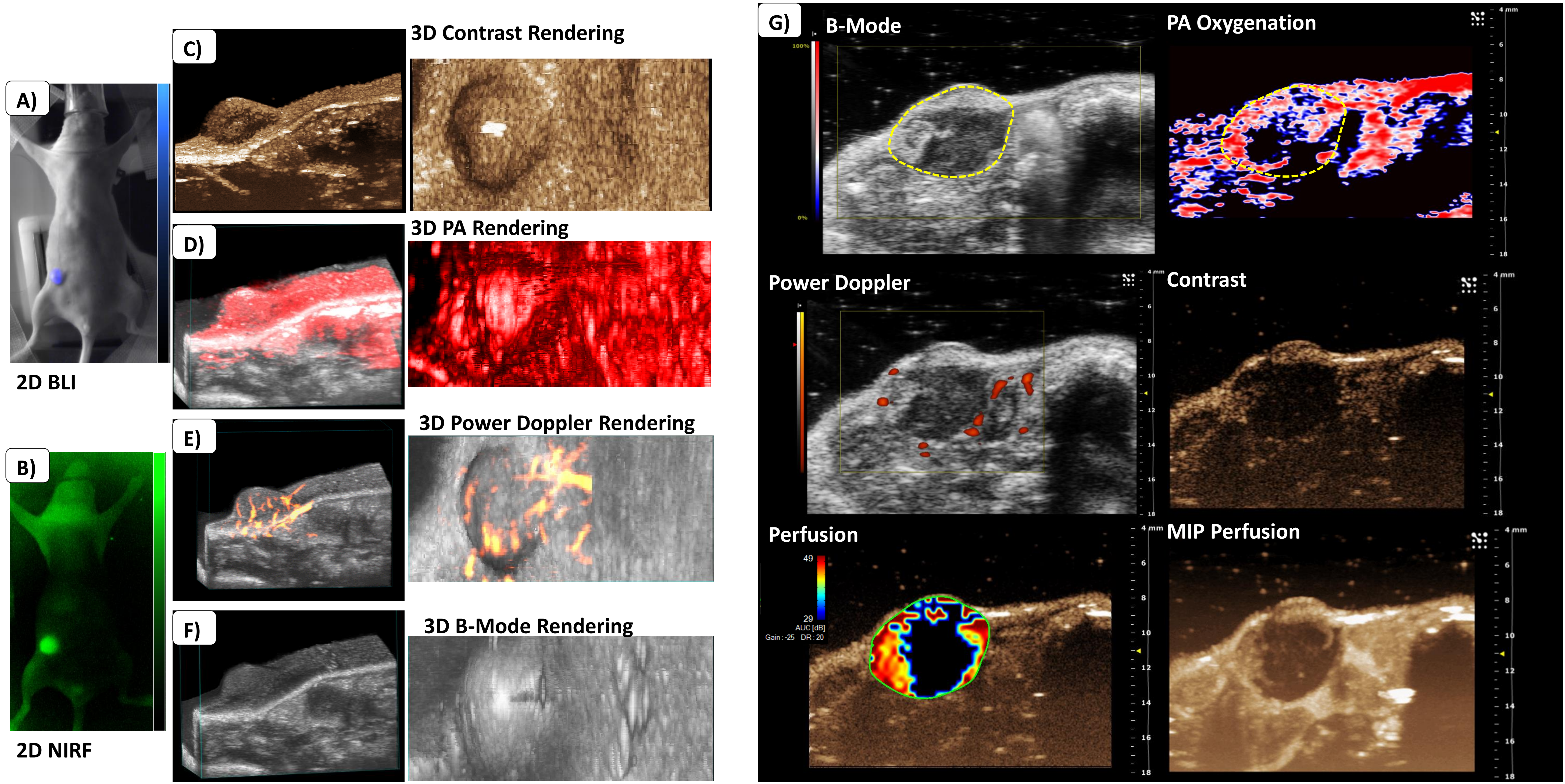
A)
2D BLI
B)
2D NIRF
C)
3D Contrast Rendering
D)
3D PA Rendering
E)
3D Power Doppler Rendering
F)
3D B-Mode Rendering
G)
B-Mode
PA Oxygenation
Power Doppler
Contrast
Perfusion
MIP Perfusion
49
29
AUC [dB]
Gain : -25 DR : 20

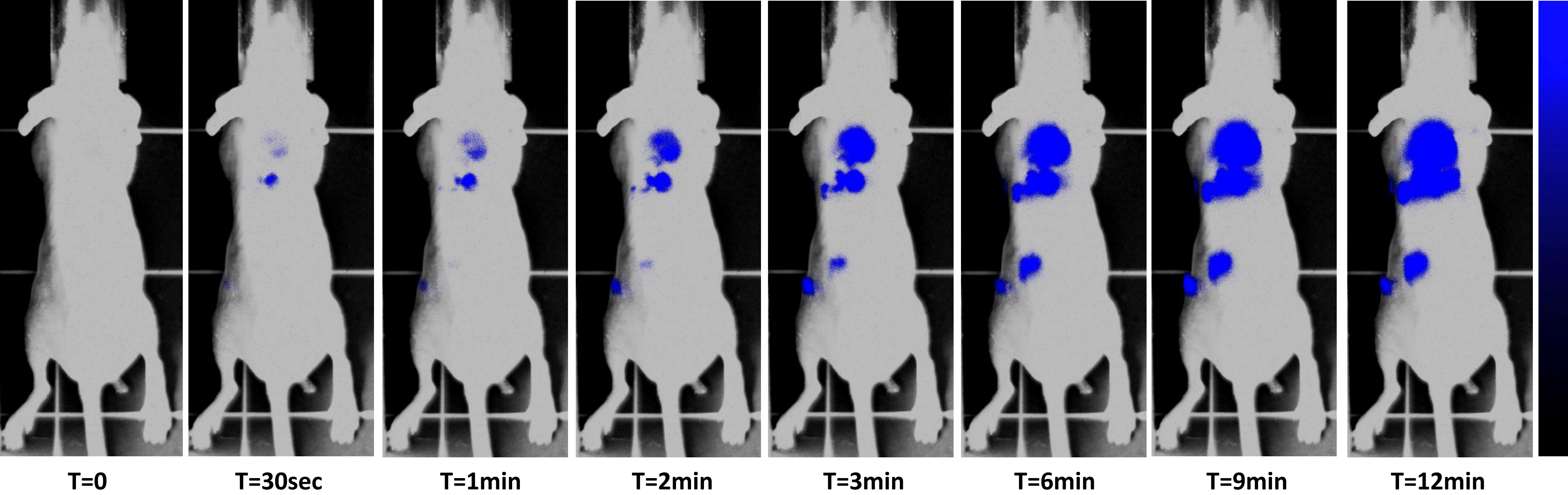
T=0
T=30sec
T=1min
T=2min
T=3min
T=6min
T=9min
T=12min
